# Probing the Role of a Non‐Thermal Plasma (NTP) in the Hybrid NTP Catalytic Oxidation of Methane

**DOI:** 10.1002/anie.201703550

**Published:** 2017-07-06

**Authors:** Emma K Gibson, Cristina E Stere, Bronagh Curran‐McAteer, Wilm Jones, Giannantonio Cibin, Diego Gianolio, Alexandre Goguet, Peter P. Wells, C. Richard A. Catlow, Paul Collier, Peter Hinde, Christopher Hardacre

**Affiliations:** ^1^ UK Catalysis Hub Research Complex at Harwell Rutherford Appleton Laboratory Harwell Oxon Didcot OX11 0FA UK; ^2^ Department of Chemistry University College London 20 Gordon Street London WC1 0AJ UK; ^3^ School of Chemical Engineering & Analytical Science University of Manchester The Mill (C56) Sackville Street Manchester M13 9PL UK; ^4^ School of Chemistry and Chemical Engineering Queen's University Belfast Belfast BT9 5AG N. Ireland UK; ^5^ Cardiff Catalysis Institute School of Chemistry Cardiff University Cardiff CF10 3AT UK; ^6^ Diamond Light Source Harwell Science and Innovation Campus Chilton, Didcot OX11 0DE UK; ^7^ School of Chemistry University of Southampton Southampton SO17 1BJ UK; ^8^ Johnson Matthey Technology Centre Reading UK

**Keywords:** EXAFS spectroscopy, heterogeneous catalysis, methane, non-thermal plasma, oxidation

## Abstract

Three recurring hypotheses are often used to explain the effect of non‐thermal plasmas (NTPs) on NTP catalytic hybrid reactions; namely, modification or heating of the catalyst or creation of new reaction pathways by plasma‐produced species. NTP‐assisted methane (CH_4_) oxidation over Pd/Al_2_O_3_ was investigated by direct monitoring of the X‐ray absorption fine structure of the catalyst, coupled with end‐of‐pipe mass spectrometry. This in situ study revealed that the catalyst did not undergo any significant structural changes under NTP conditions. However, the NTP did lead to an increase in the temperature of the Pd nanoparticles; although this temperature rise was insufficient to activate the thermal CH_4_ oxidation reaction. The contribution of a lower activation barrier alternative reaction pathway involving the formation of CH_3_(g) from electron impact reactions is proposed.

Methane is a major contributor to climate change, with a global warming potential at least 21 times higher than that of CO_2_; consequently, its release into the atmosphere must be stringently controlled. In addition to controlling CH_4_ release from landfills, biomass burning, and leakage from natural gas storage and distribution, emission abatement arising from CH_4_ slip in automotive vehicles must also be addressed.

One solution is to use catalytic total oxidation to produce CO_2_ and water. Palladium is a known efficient catalyst for CH_4_ oxidation and has been studied extensively. Varying hypotheses of the active phase have been reported, from a PdO‐like phase to Pd^0^.^1^ Unfortunately, of all the catalysts currently reported, none are sufficiently active under cold‐start conditions, with most catalysts requiring light‐off temperatures of around 400 °C.^2^ Such high temperatures are required because of the high activation barriers to CH_4_ dehydrogenation; particularly formation of surface adsorbed CH_3_* and H*, which is thought to be the rate‐determining step. For example, Jørgensen and Grönbeck predicted that the extraction of the first H from CH_4_ had activation barriers of 0.99 and 0.79 eV over Pd(111) and Pd(100), respectively.^3^ One known method for inducing catalytic activity in kinetically restricted reactions at low temperatures is by coupling non‐thermal plasmas (NTPs) with catalysis. Recent examples are the selective catalytic reduction of NO_*x*_, volatile organic carbon (VOC) removal, and water gas shift without the need for an external heating source. Similarly, NTP‐assisted CH_4_ oxidation has been reported at low temperature, where no additional heat source is applied, and at elevated temperatures, where the catalyst is also heated to temperatures up to 300 °C.^4^

Three recurring hypotheses are often proposed to explain the assistance which NTP gives to catalytic reactions: 1) the plasma modifies the catalyst, 2) the plasma heats the catalyst, and 3) the assistance of the plasma permits occurrence of new reaction pathways. NTP has been shown, in some cases, to alter the catalysts itself by changing the oxidation state^5^ or metal surface area^6^, ^7^ of the components. Several attempts have been made to determine the temperature of a catalyst during NTP reactions, using thermocouples placed near or in the catalyst bed, or by observation with infrared cameras or optical emission spectroscopy.^8^ However, no study has yet directly measured the temperature of the metal nanoparticles within the catalyst and compared this with the overall bed temperature during a NTP‐assisted catalytic reaction. The interaction of radicals, electrons, or photons produced by the NTP with the catalyst and the adsorbed molecules may open up new reaction pathways. For instance, the direct reaction of gas‐phase radicals with adsorbed species (that is, a direct Eley–Rideal mechanism) could occur.^9^ Desorption from the catalyst surface may also be aided by electron impact.^10^ To investigate which of these hypotheses are operating under NTP‐assisted catalysis, in situ investigations are crucial. Very few in situ plasma catalytic studies have been performed.^11^ One recent example involved investigation of the hydrocarbon selective catalytic reduction (HC‐SCR) of NO_*x*_ over Ag/Al_2_O_3_.^12^ This investigation used a modified diffuse reflectance infrared Fourier transform (DRIFTS) setup to follow the adsorbates during the thermal and the NTP‐enhanced reactions; providing invaluable information on adsorbed species and the mechanism of the reaction. However, to the best of our knowledge, no in situ structural studies have been undertaken to characterize the catalyst during NTP‐assisted reactions. In the study reported herein, we have probed NTP‐assisted CH_4_ oxidation, in the absence of any applied external heating, using in situ X‐ray absorption fine structure (XAFS) information. The results provide significant new insights into the role of plasma‐induced heating effects in the NTP‐assisted process.

The NTP‐activated oxidation of CH_4_ over a 2 % Pd/Al_2_O_3_ (sample 1; Supporting Information, Table S1) was examined at applied voltages of 6 and 7 kV at 22.5 kHz. In the absence of an externally applied heating source, 55 % (6 kV) and 67 % (7 kV) CH_4_ conversions were observed. In the presence of the catalyst, high selectivities were found (CO:CO_2_, 1:10.8 (6 kV) and 1:11.5 (7 kV)) and negligible amounts of H_2_ were formed. Additionally, a temperature‐programmed oxidation measurement of the catalyst following CH_4_ oxidation showed little carbon deposition with no significant oxidation above >600 °C (Supporting Information, Figure S1). Notably, in the absence of the catalyst, although the conversion was similar (68 % at 7 kV), significantly more CO was formed (CO:CO_2_, 1:1.1). These results may also be compared with the reaction over the discrete support in the presence of the plasma, which had a reduced conversion of 59 % and a CO:CO_2_ ratio of 1:2.1. These observations demonstrate the importance of the catalyst in determining the selectivity of the reaction. The specific energy input (SEI) calculated for the 6 and 7 kV plasma in the presence of the catalyst was 2.133 kJ L^−1^ and 2.637 kJ L^−1^, respectively.

Similar conversions/selectivities were also obtained during the X‐ray absorption spectroscopy (XAS) investigation monitored by end‐of‐pipe mass spectrometry (MS) analysis (sample 1, 52 % conversion at 6 kV; Supporting Information, Figure S2). The setup, shown in Figure 1 and Figure S3 (Supporting Information), allowed XAFS measurements along the length of the packed catalyst bed to be monitored. XAFS measurements were performed at the Pd K‐edge on the B18 beamline at the Diamond Light Source, United Kingdom.

**Figure 1:**
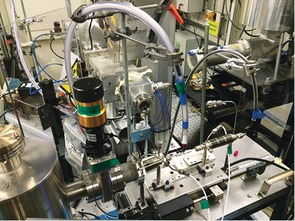
Setup for in situ measurements using XAFS spectroscopy coupled with MS for CH_4_ oxidation using plasma.

The X‐ray absorption near‐edge structure (XANES) of the fresh catalyst (sample 1) under thermal CH_4_ oxidation reaction (352 °C) compared to that under CH_4_ oxidation using the plasma are very similar, as shown in Figure 2. On first inspection, there is little impact of the plasma on the catalyst, with the spectra closely resembling that of PdO. When oxygen is removed from the system under plasma, leaving just 5000 ppm CH_4_ and He balance, the catalyst is reduced, as shown by a shift in the edge position of 2 eV, and the spectra are very similar to that of the Pd foil reference. Analysis of the extended X‐ray absorption fine structure (EXAFS) region reveals only very subtle differences in the spectra when comparing the plasma‐activated and thermally activated catalysts under CH_4_ oxidation conditions. Spectra collected at two positions, 2.5 and 7.5 mm from the start of the catalyst bed, are shown in Figure 3 and the fitting parameters are shown in Table 1. In both cases the oscillations are dampened when the plasma is on compared to when it is off. No other differences are observed in the spectra; for example no shift in phase or additional features are observed that would indicate changes in distance to nearest neighbors or changes in the coordination around the absorber atom. Furthermore, on turning off the plasma, no change was found compared with the fresh catalyst (Supporting Information, Figure S4), demonstrating the reversibility of the changes and indicating that no significant permanent changes to the nanoparticle structure had occurred. Notably, XAFS is a bulk‐averaging technique; therefore, we cannot exclude that some minor non‐reversible changes to the Pd nanoparticles may occur, which are below the detection level of the technique. However, the EXAFS results are consistent with transmission electron micrographs (TEM) of the catalyst before and after the plasma treatment, which showed similar particle sizes 2.1±1.0 and 2.9±1.4 nm and no change in the shape of the nanoparticles observed (Supporting Information, Figure S5).

**Figure 2:**
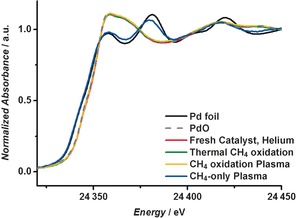
Normalized XANES spectra of the Pd foil reference, the PdO reference, the fresh catalyst (sample 1) under helium gas, the catalyst during the thermal CH_4_ oxidation reaction, the catalyst during plasma‐activated CH_4_ oxidation, and during CH_4_‐only reaction under plasma conditions.

**Figure 3:**
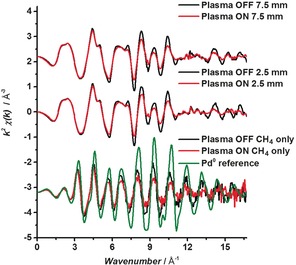
*k* ^2^ *χ*(*k*) data for CH_4_ oxidation at the start (position 2.5) and middle (position 7.5) of the catalyst (sample 1) bed, under plasma ON and plasma OFF conditions, and the CH_4_‐only experiment under plasma ON and OFF conditions.

**Table 1:** EXAFS Fitting Parameters for Pd K‐Edge spectra taken under CH_4_ oxidation and CH_4_‐only conditions for sample 1.

Conditions	Absorber– Scatterer	Δ*E*_0_ [eV]	CN^[c]^	*R*_eff_ [Å]	*σ* ^2^	*σ* ^2^ (plasma ON)	*T* [°C] (plasma ON)
^[a]^CH_4_ oxidation, plasma OFF, front 2.5 mm)	Pd_O	‐0.7(8)	4.2(6)	2.024(6)	0.0025(6)	0.0043(5)	203±32
	Pd_Pd1		4.2(6)	3.058(6)	0.0059(8)	0.0099(7)	
	Pd_Pd2		4.9(8)	3.445(6)	0.0054(9)	0.0095(9)	

^[a]^CH_4_ oxidation, plasma OFF, middle (7.5 mm)	Pd_O	3.9(2)	3.9(2)	2.026(5)	0.0022(5)	0.0038(4)	152±28
	Pd_Pd1		4.3(6)	3.059(6)	0.0059(7)	0.0088(6)	
	Pd_Pd2		4.7(8)	3.449(6)	0.0053(8)	0.0092(8)	

^[b]^CH_4_ coupling, plasma OFF, middle (7.5 mm)	Pd_Pd1	5(1)	7.5(5)	2.76(9)	0.0057(2)	0.0097(7)	207±32
	Pd_Pd2		2(2)	3.8(6)	0.0081(1)	0.0158(9)	

[a] Fitting parameters: *S*_0_^2^=0.85, as determined by the use of a Pd foil standard. Fit range 3.0<*k*<16.0, 1.0<*R*<3.5; number of independent points=20.5. [b] Fitting parameters: 1.2<*R*<4, 3.4<*k*<10.6; number of independent points=12.6.

We propose that the subtle dampening of the oscillations is due to an increase in the temperature of the Pd under the application of the plasma, when an increase in the mean‐squared thermal disorder parameter (*σ* ^2^) corresponds to a decrease in the amplitude of the EXAFS oscillations. Similarly, weak oscillations were also observed for a range of catalyst loadings and particle sizes (samples 1–4; Supporting Information, Table S1) under plasma conditions. As no other measurable changes occur to the catalyst, the change in *σ* ^2^ can be used to estimate the temperature of the Pd nanoparticles on the catalyst. A data set was obtained on cooling of the catalyst from 500 °C to room temperature under air, which indicated no structural changes on cooling and was used to determine a calibration curve of the variation of *σ* ^2^ with temperature.

The calibration curve and fitting parameters are shown in the Supporting Information, Figures S6–S9 and Tables S2 and S3. These data were then used to determine the temperature of the catalysts when activated by the plasma. To fit the data upon cooling, the EXAFS fit was performed, allowing the coordination numbers (CN), σ^2^ values, and distances to refine. The determined CN values were then fixed when fitting the plasma‐activated spectrum, allowing only *σ* ^2^ and distances to refine. This value was then used to estimate the temperature of the catalyst when activated by the plasma (Supporting Information, Table S4). For all the catalysts studied the estimated temperatures ranged from 138 to 179 °C in the middle of the bed. Additionally, measurements were made for sample 1 comparing the front and middle of the bed, and the temperatures were determined to be 203±32 and 152±28 °C, respectively. The fitting parameters (Table 1) and the data and fit are shown in Figure S7 (Supporting Information). The higher temperature at the front of the bed is expected as the CH_4_ oxidation is exothermic and the rate will be highest towards the start of the catalyst bed. Using Aspen software (Aspen Technology), a simulation of the reaction using the same reactant concentrations and assuming thermodynamic equilibrium (that is, full CH_4_ conversion) provided a reactor temperature of approximately 210 °C, which is consistent with the exothermicity of the CH_4_ oxidation.

The estimated temperatures of the catalyst bed were significantly higher than those measured using an infrared (IR) camera, Figure 4 (60–90 °C), which is a technique commonly used to determine plasma reaction temperatures.^8^ This measurement is not surprising as the IR camera predominantly measures the outside wall of the reactor and will significantly underestimate the temperature within the packed bed.

**Figure 4:**
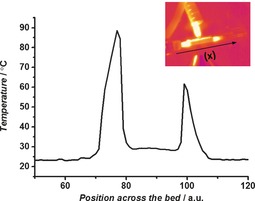
Temperature profile along the catalyst bed (*x* direction) using an IR camera; the units of *x* are arbitrary and are dependent on the camera focus.

To determine if the observed heating of the catalyst was because of the exothermicity of the CH_4_ oxidation reaction or induced by the plasma, the reaction was performed in the absence of O_2_. Under these conditions the reaction of CH_4_ is endothermic and results in coupling products.^13^ During the endothermic non‐oxidative CH_4_ coupling, the EXAFS data (Figure 2, Table 1) obtained when the plasma is both on and off resembles that of the Pd foil; therefore, the PdO catalyst has been, unsurprisingly, reduced on removal of oxygen from the feed gas. Using the value of *σ* ^2^ calculated from this data, the estimated temperature during the plasma was 207±32 °C (Table 1). From these results we conclude that the plasma is responsible for the observed heating effect.

The fact that there are no significant nanoparticle size‐ or catalyst loading‐dependent changes on the temperatures calculated from the XAFS may suggest that the surface of the whole catalyst (nanoparticle and oxide) is being heated. The XAFS data only probes the Pd, which, however, does not preclude the alumina surface from also being heated. In this case, the support and nanoparticle would be in thermal equilibrium, thereby leading to similar changes in temperature for all the catalysts studied.

Taking account of all the data acquired, the estimated temperature of the nanoparticles during NTP‐activated CH_4_ oxidation is 162±24 °C, which is within the error of the calculated temperature from the exothermicity of the CH_4_ oxidation reaction. Therefore, a clear difference in temperature is observed between the EXAFS estimation and that measured by the IR camera. Almost a two‐fold increase in temperature of the nanoparticles is measured compared to the overall temperature of the catalyst bed. However, this temperature (162 °C) is not high enough to activate the thermal reaction, as observed from the light‐off curve of the thermal reaction for sample 1 (Figure 5).

**Figure 5:**
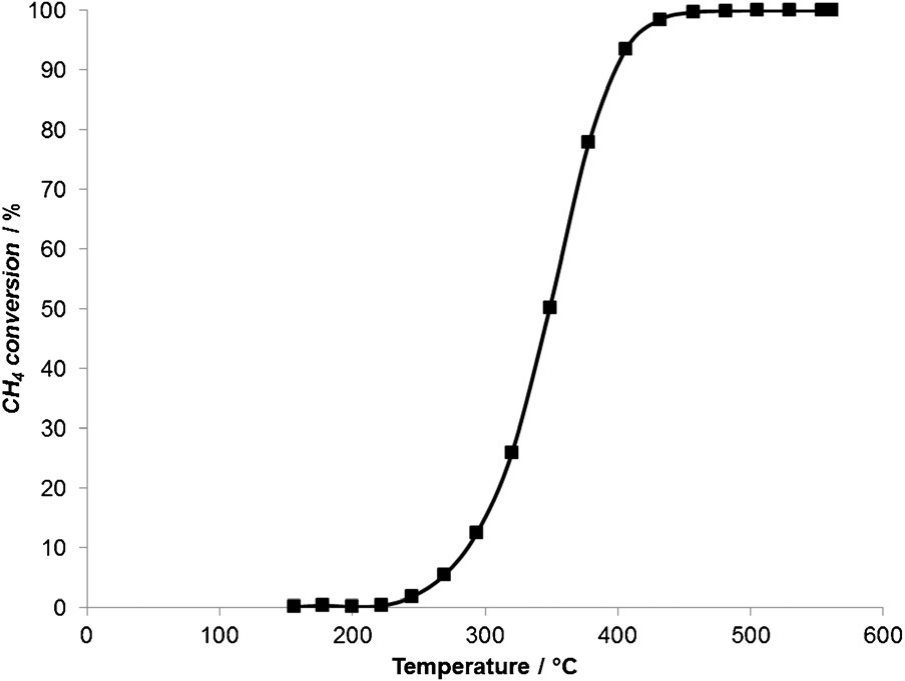
Light‐off profile 5000 ppm CH_4_, 5 % O_2_, 5 % Ar, and He balance.

In summary, this in situ study has provided evidence for the role of NTP in the hybrid NTP catalytic oxidation of CH_4_. Herein, it is clear that no significant structural changes are found within the catalyst on application of NTP under CH_4_ and CH_4_+O_2_ conditions. Additionally, the NTP heats the Pd nanoparticles but the temperature of the nanoparticles is lower than that necessary to activate the thermal CH_4_ oxidation reaction. Therefore, it is likely that an alternative CH_4_ activation pathway is in operation, with a lower activation barrier than the thermal activation reaction. As noted, the rate‐limiting step for the thermal reaction over Pd is the formation of CH_3(a)_+H_(a)_. This is found above 227 °C, whereas carbon oxidation is rate limiting below 227 °C.^3^ Interestingly, the major effect of the plasma on CH_4_ has recently been reported to be the formation of CH_3_(g) by electron impact reactions.^14^ Given the fact the nanoparticle temperatures are approximately at the transition point where CH_4_ activation becomes rate limiting, it is likely that the plasma activation of CH_4_ in the gas phase then leads to a reduced activation barrier for the surface process and thus the ability of the NTP process to occur at much reduced temperatures. It cannot be discounted that the Pd nanoparticles may become more defective in the presence of the plasma and more open faces have been reported to offer a lower activation barrier for CH_4_ dehydrogenation.^3^ However, this effect is likely to be small compared with the preactivation of CH_4_ in the gas phase under plasma conditions.

## Conflict of interest

The authors declare no conflict of interest.

## Supporting information

As a service to our authors and readers, this journal provides supporting information supplied by the authors. Such materials are peer reviewed and may be re‐organized for online delivery, but are not copy‐edited or typeset. Technical support issues arising from supporting information (other than missing files) should be addressed to the authors.

Supplementary
