# Supplementary material for: Probing the Role of a Non‐Thermal Plasma (NTP) in the Hybrid NTP Catalytic Oxidation of Methane
Source: Angew Chem Int Ed Engl. 2017 Jul 6;56(32):9351–5. doi: 10.1002/anie.201703550 (PMC5577514; doi:10.1002/anie.201703550)
Supplement: Supplementary file 1 — Supplementary [file ANIE-56-9351-s001.pdf]

Angewandte Chemie
Eine Zeitschrift der Gesellschaft Deutscher Chemiker

Supporting Information

Probing the Role of a Non-Thermal Plasma (NTP) in the Hybrid NTP Catalytic Oxidation of Methane

Emma K Gibson, Cristina E Stere, Bronagh Curran-McAteer, Wilm Jones, Giannantonio Cibin, Diego Gianolio, Alexandre Goguet,* Peter P. Wells, C. Richard A. Catlow, Paul Collier, Peter Hinde, and Christopher Hardacre**

anie_201703550_sm_miscellaneous_information.pdf

Electronic Supplementary Information

Catalytic activity with conventional heating or plasma

The catalyst (250 - 425μm particle size) was held in place between quartz wool plugs in a 6mm OD quartz tube with a K-type thermocouple placed in the centre of the catalyst bed to monitor the reaction temperature during light-off experiments. The gases in the feed were controlled individually using mass flow controllers (Area, Advanced Energy). The gas exiting the reactor was analysed using a gas chromatograph (Agilent 7820A) equipped with a Parapak Q packed column and a flame ionisation detector (FID) fitted with a methanizer to allow measurement of CO and CO_2.

The plug-flow reactor was adapted for plasma-generation by replacing the internal thermocouple with a 0.5 mm O.D. tungsten wire that acted as a ground electrode and a stainless steel mesh on the outer of the reactor (anode). In order to prevent arcing due to increase thermal conductivity of the dielectric medium in the presence of water, insulation of the ground electrode was achieved by enclosing the tungsten wire into a 1.8 mm sealed quartz capillary. The plasma was ignited by using an alternating current power source (PVM500 model) and the electrical parameters were monitored using an oscilloscope (Tektronix TBS1072B) that was connected to the reactor through a high voltage probe (Tektronix, P6015). The applied voltages were 6.0 and 7.0 kV at frequencies between 23-30 kHz. The catalyst was carefully packed in the dielectric barrier discharge region to ensure a direct contact with the plasma-generated species and to take advantage of any possible local heating effects.

The feed gas stream consisted of 5000 ppm CH_4, 5% O_2 and Ar balance. The total flow rate and space velocity of the gas mixture were 200 cm^3min^{-1} and 120,000 $cm^3g^{-1}h^{-1}$, respectively.

Thermally activated light off tests were carried out by increasing the reaction temperature at 1 °C min^{-1} from 100 °C to 560 °C.

Temperature programmed oxidation (TPO) measurement using Hiden HPR20 mass spectrometer were performed after ~60 min reaction to investigate and remove carbonaceous species and other species that can be oxidised by O_2. Approximately 50 mg of catalyst was exposed to a gas mixture of 5%O_2/0.5%Kr/Ar with a total flow rate of 100 cm^3 min^{-1} for 10 min at 40 °C in order to gain the stabilised baselines of all species that need to be monitored. After that, the temperature was increased at a controlled rate of 10 ºC min^{-1} up to 750 ºC. The evolving gases such as CO, CO_2, O_2 and H_2O were monitored as a function of temperature. The data for the TPO following the NTP assisted methane combustion is shown in Figure S1.

In-situ XAS under thermal and plasma activation

The setup, shown in Figure S3, allowed XAFS spectra measurements along the length of the packed catalyst bed to be monitored. XAFS measurements were performed at the Pd K-edge on the B18 beamline at the Diamond Light Source, Didcot, UK. The dielectric barrier discharge was generated using an AC power supply (Model PVM500). The power electrode, a tungsten wire (0.125 mm I.D.) placed inside a quartz capillary fused at one end (0.175 mm I.D.), was inserted along the centre of the catalyst bed, which itself was packed inside a

6mm O.D. quartz tube held in place using glass wool. Aluminium foil was used as the ground electrode, wrapped around the outside of the reactor tube covering the area of the catalyst bed (~8.5 mm in length). This afforded good transmission of the X-rays through the catalyst bed. The power electrode was driven at ~21 kHz with peak applied voltages of 6 kV. The total flow rate and space velocity of the gas mixture were 200 cm^3 min^{-1} and 120,000 cm^3 $g^{-1}h^{-1}$, respectively. The concentrations of reactants were 5000 ppm CH_4, 5% O_2 (when used), 5% Ar and He balance. The Ar was present to provide a constant reference for the signal in the mass spectrometer. It should be noted that there was no significant effect of the presence of the Ar, the conversions in He and in Ar were similar under the NTP conditions (Figure S2). The same reactor was used for the XAS measurements under thermal conditions with a K-type thermocouple placed in the catalyst bed. Heating was applied using a hot air blower. The calculation for the theoretical temperature rise was calculated using Aspen Plus V8.4 (Aspen Technology). It was performed using experimental molar flow rate and an RGibbs reactor in adiabatic mode. The exotherm is therefore a worst case scenario assuming an adiabatic temperature increase.

XAFS measurements were performed at the Pd K-edge on the B18 beamline at the Diamond Light Source, Didcot, UK. Measurements were performed in transmission mode using a QEXAFS setup with fast-scanning Si(311) double crystal monochromator. XAFS spectra were acquired in 60 s and averaged over at least 5 spectra (k_{max} = 19 Å), concurrently with the appropriate foil placed between I_t and I_{ref}. XAFS data processing was performed using IFEFFIT with the Horae package (Athena and Artemis).[2,3] The data collected was fitted using 3 scattering paths for PdO, Pd_Pd, Pd_O and a longer Pd_Pd path.

The power measurements were undertaken using the resistor method, which implies using a resistor with known resistance (R = 52 Ω) placed in series between the power electrode and the ground. Typical current-voltage signals are shown in Figure S3(d). Using equation 1, the current (I) is calculated from the measured voltage across the resistor (V_R). The averaged power (P) is calculated by averaging the instantaneous power over one cycle (period) T as described in reference 1, using equation 2. The specific energy input (SEI) was calculated using equation 3, where Q_{gas} is the gas flow rate (L s^{-1}).

$$\mathrm{I}\,(A) = \frac{V_R}{\mathrm{R}} \qquad \textit{Equation 1}$$

$$P(W) = \frac{1}{\mathrm{T}}\int_0^{\mathrm{T}} \mathrm{I(t)}.\mathrm{V(t)}\,\mathrm{dt} \qquad \textit{Equation 2}$$

$$SEI\,\left(\frac{J}{L}\right) = \frac{\mathrm{P}}{\mathrm{Qgas}} \qquad \textit{Equation 3}$$

Four samples were used during this investigation, listed in Table S1. Sample 1 was used for the majority of the NTP reactions, those shown in Figures 2, 3, 4, 5, the TPO study and the comparison of activity at differing NTP peak Voltages. Sample 3 was used for the calibration study of σ^2 vs. temperature, outlined below. Samples 1-4 were used to investigate the effect of nanoparticle size and Pd loading on the measured nanoparticle temperature.

Transmission electron microscopy (TEM) samples were prepared by dispersing the catalyst powder in high purity ethanol, followed by sonication for 10 min. A drop of this suspension was then evaporated on a holey carbon film supported by a 300 mesh copper TEM grid. Samples were then subjected to bright field diffraction contrast imaging to determine particle size distribution. The TEM instrument used for this analysis was a JOEL-2100 with a LaB6 filament operated at 200 kV.

Table S1. Catalyst sample list and description.

Sample	Size (nm)	Pd loading (wt%)
1	2.1	2
2	4.0	2
3	1.6	3
4	0.8	3

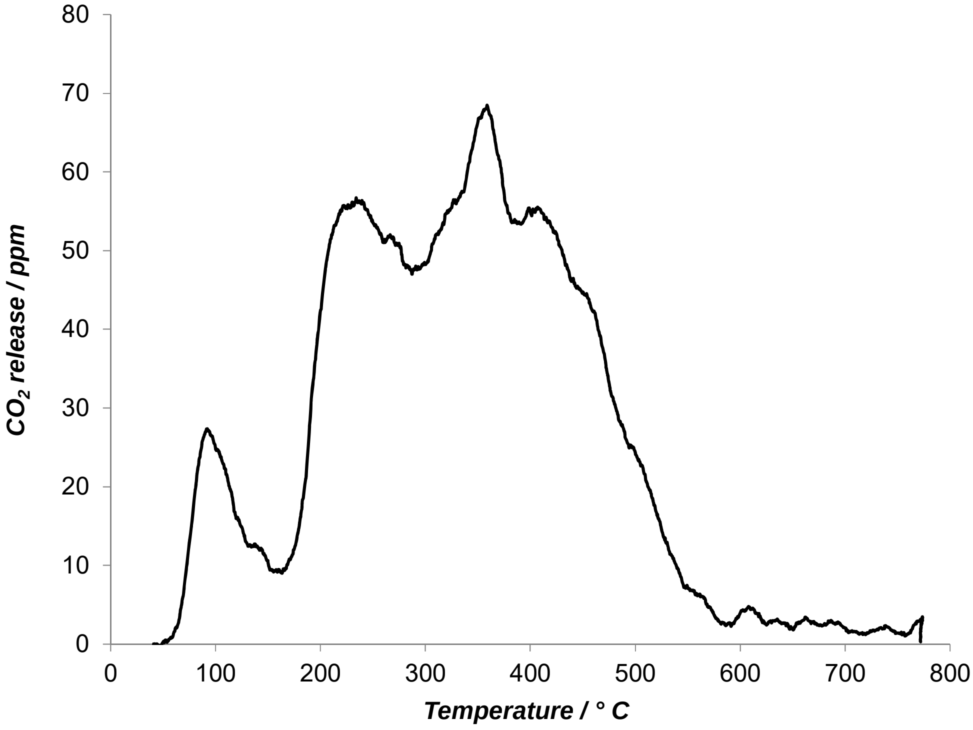

Figure S1 TPO profile of 2% Pd/Al_2O_3 catalyst (sample 1) following a NTP activated CH_4 oxidation reaction at 6 kV for 60 min.

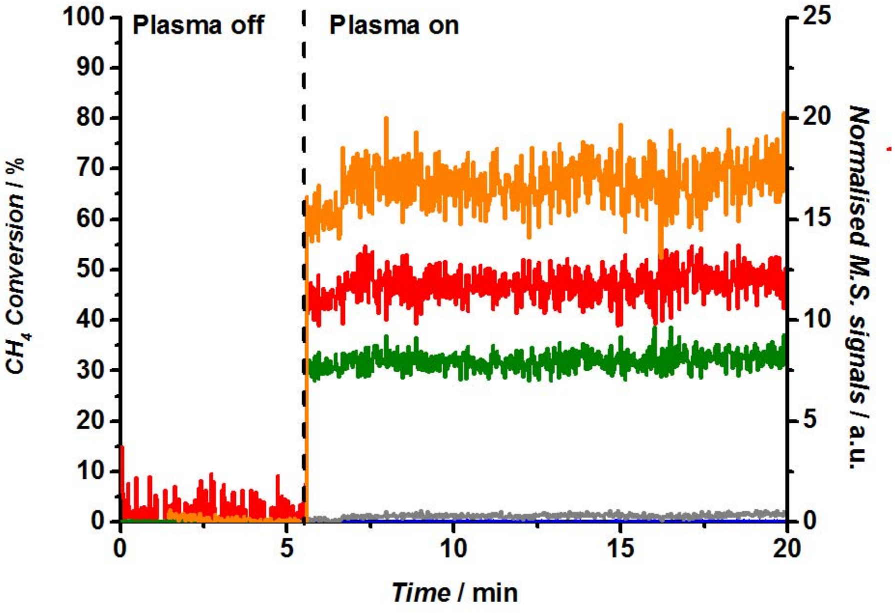

Figure S2 Mass spectrometry results for plasma off-plasma on experiment, 5000 ppm CH_4, 5% O_2, 5% Ar and He balance over sample 1; CH_4 conversion (red) and the mass spectrometry responses of CO, H_2O, H_2 and CO_2 are grey, orange, blue and green, respectively.

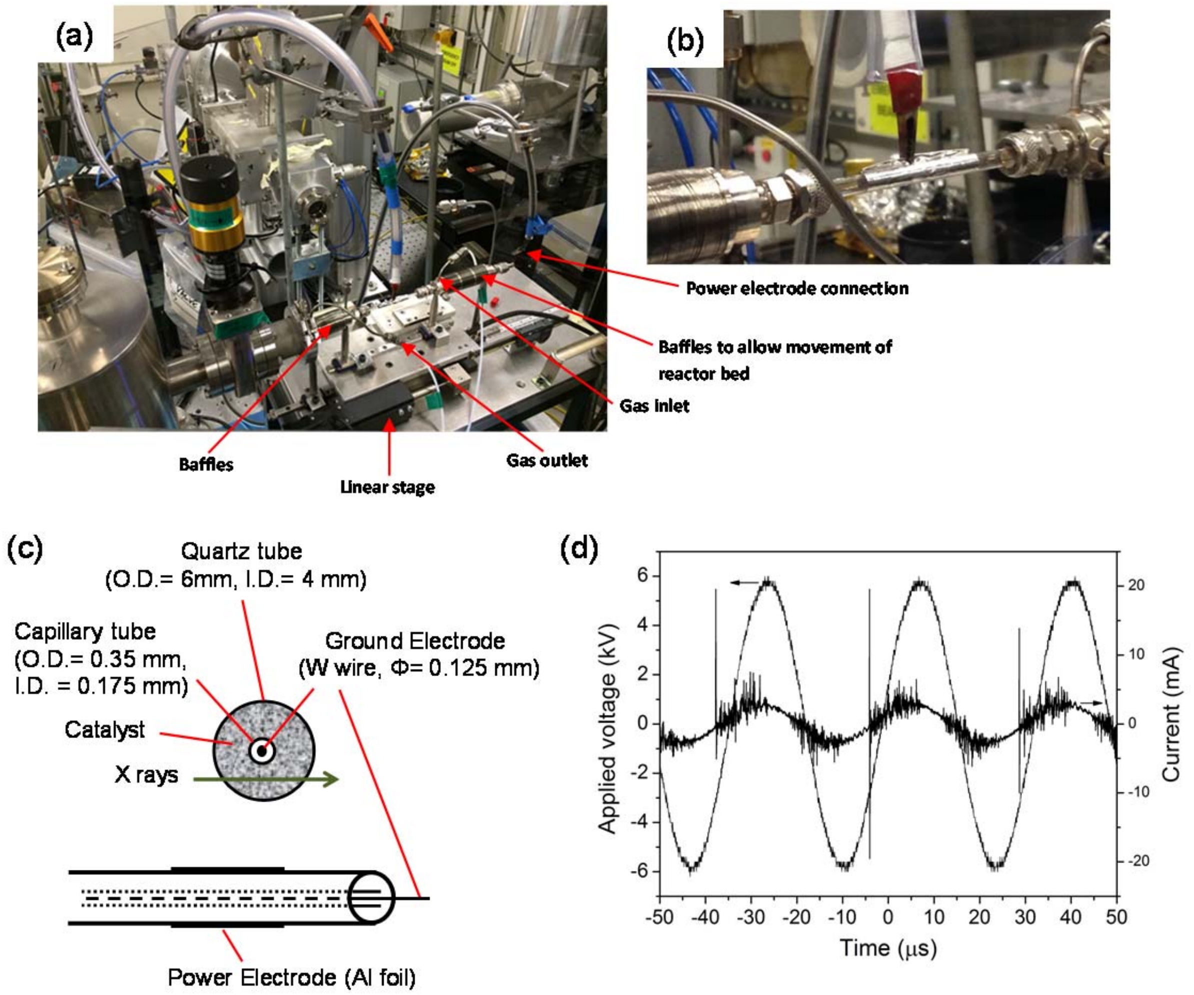

Figure S3 (a) Setup for *in situ* measurements using XAFS spectroscopy coupled with mass spectrometry for methane oxidation using plasma, (b) zoomed image of reactor tub with connected aluminium foil ground electrode, (c) schematic of the reactor showing the optical path of the X-rays and (d) current-voltage measured waveform profiles.

Reversible effect of NTP on the size and structure of the catalyst

The k^2 weighted χ data of the catalyst before, during and after non-thermal plasma assisted methane oxidation is shown in Figure S4. The plasma off data recorded before and after the plasma assisted reaction overlay well, showing no shift in phase or additional features, confirming the reversibility of the changes to the nanoparticles during reaction, i.e. that the NTP has not permanently changed the Pd nanoparticles.

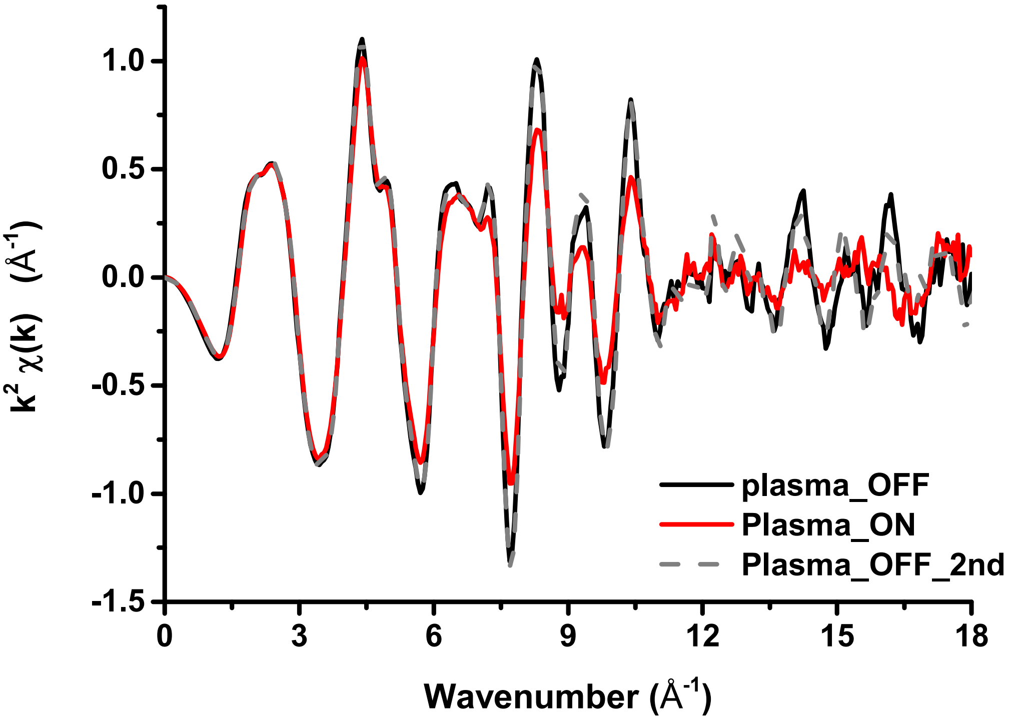

Figure S4 $k^2\chi$ data of the Pd/Al_2O_3 catalyst (Sample 1) before plasma (black line), after NTP-CH_4 oxidation (red line) and after the plasma was switched off (grey dashed line).

Electron Micrographs of the effect NTP on the size and structure of the catalyst

The TEM data recorded for the fresh catalyst (Sample 1) and the post NTP CH_4 oxidation reaction have nanoparticle size distributions of 2.1 ± 1.0 and 2.9 ± 1.4 nm, respectively, Figure S5.

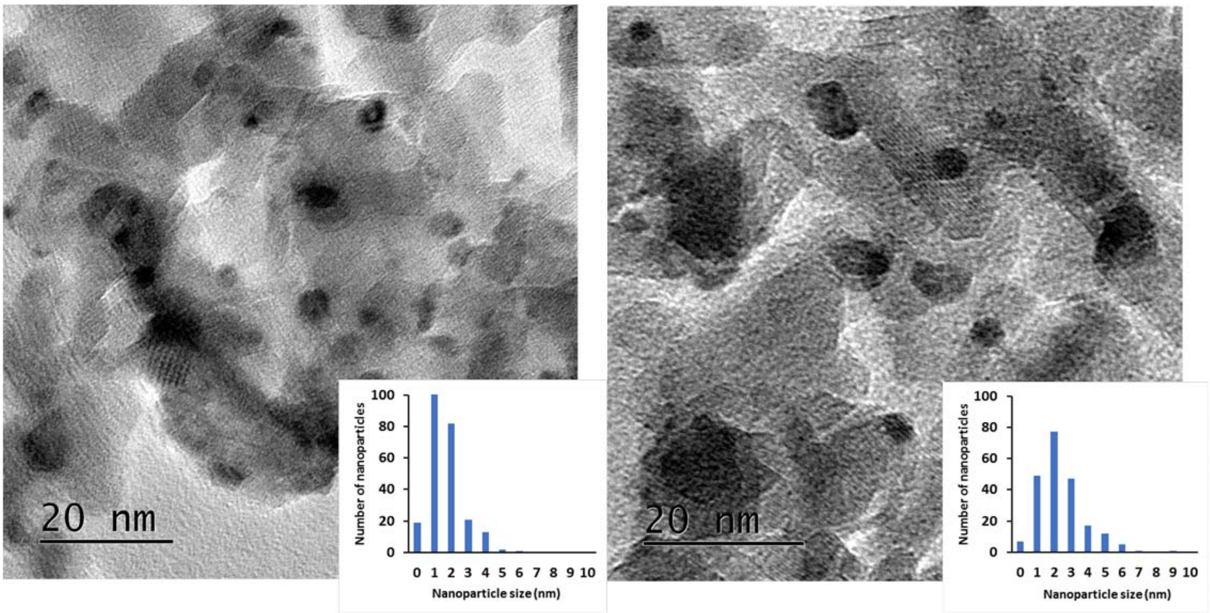

Figure S5 TEM micrographs and overlaid histograms of the fresh (left) and post NTP CH_4 oxidation (right) catalyst (Sample 1).

XAS data, fits and fitting parameters under thermal and NTP conditions

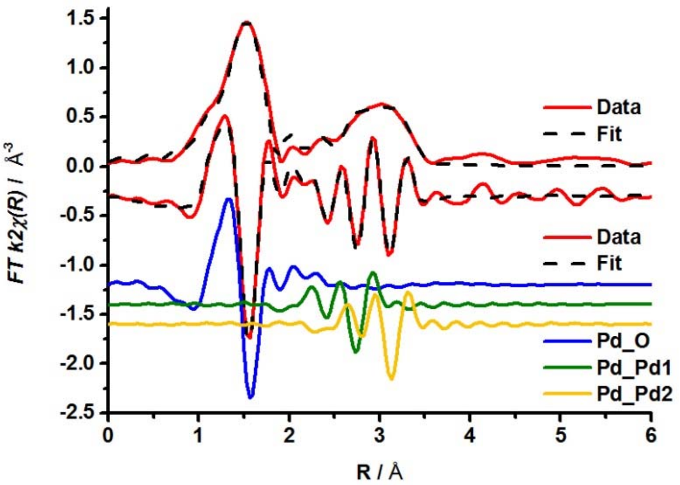

Figure S6 Pd K-edge EXAFS data at RT (sample 1) showing the magnitude and imaginary components of the k^2 weighted Fourier transform data and fits and the imaginary components of each scattering path used in the fits.

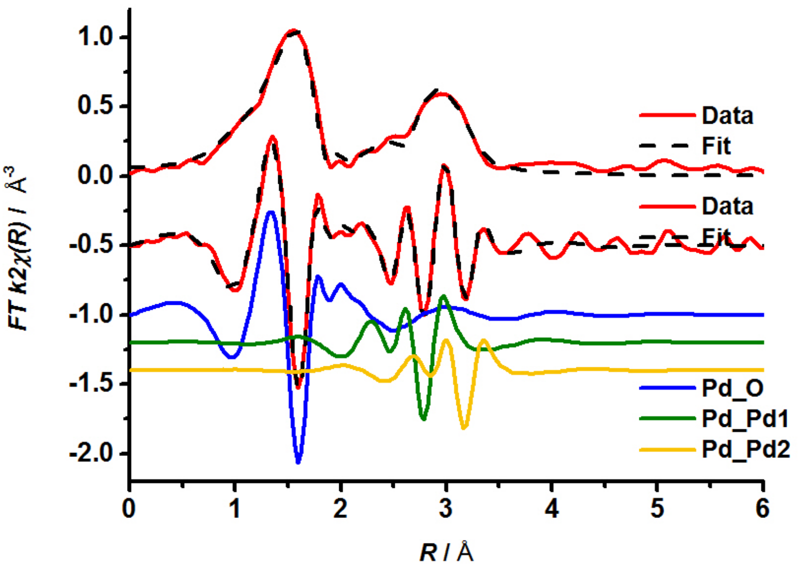

Figure S7 Pd K-edge EXAFS data recorded during NTP assisted CH_4 oxidation at position 2.5 mm into the catalyst bed (sample 1). Showing the magnitude and imaginary components of the k^2 weighted Fourier transform data and fits and the imaginary components of each scattering path used in the fits.

Determination of calibration curve for σ^2

XAFS data was recorded during the cooling in air from 500 to 25°C of a PdO/Al_2O_3 catalyst (Sample 3). As no change in the XAFS spectra were observed over this temperature range, Figure S8, it was assumed that any change in intensity of the EXAFS oscillations was an effect of temperature. The fitting parameters determined for the RT data were then fixed and only σ^2 for each path refined for the other temperatures, see Tables S2 and S3. The resulting calibration curve is shown in Figure S9. The fitting of the EXAFS data of Sample 1 'fresh' prior to NTP reactions, is shown for comparison in Table S2. The fitting results, EXAFS data and the XANES spectra shown in Figure S10 and Table S2, show that the sample used for the calibration curve, and the sample studied for the NTP reactions, both fit well to scattering paths for PdO. The refined coordination numbers for the scattering paths are also similar, indicating these samples are of similar size, as is consistent with the particle sizes listed in Table S1.

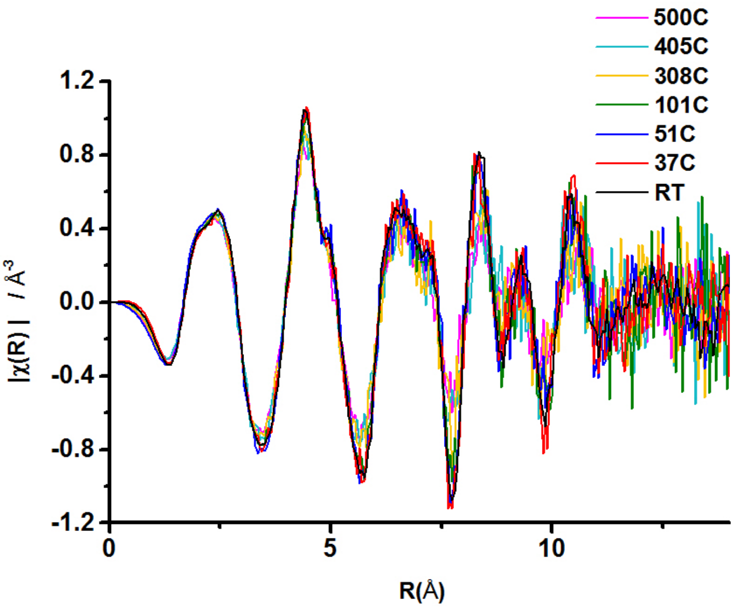

Figure S8 $k^2\chi$ data of the PdO/Al_2O_3 catalyst (sample 3) during cooling from 500°C to RT.

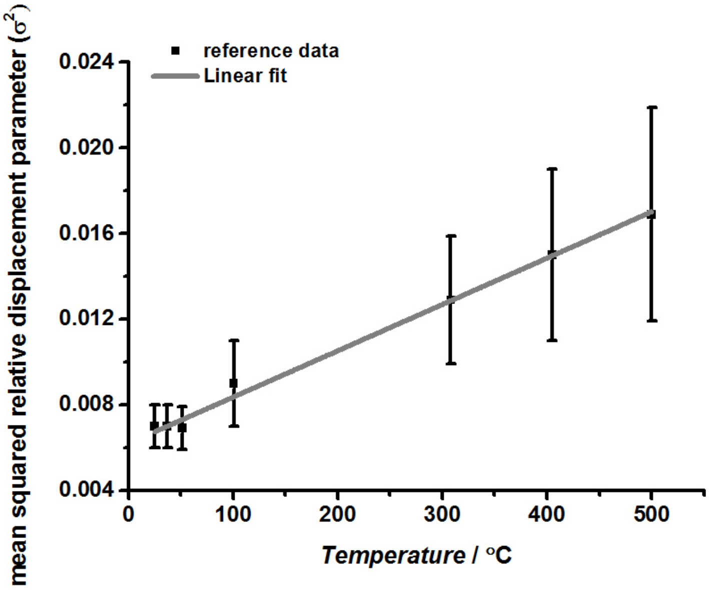

Figure S9 Calibration curve, σ^2 values vs temperature from Table S2.

Table S2. EXAFS Fitting Parameters for Pd K-Edge spectra of PdO/Al_2O_3 taken during cooling in air compared to the fresh catalyst (Sample 1) prior to NTP reaction.

Temperature	Absorber-Scatterer	ΔE_0 (eV)	CN[c]	R_{eff} (Å)	σ^2
Sample 3 at 25°C	Pd_O	4.6±0.8	3.7±0.2	2.010±0.008	0.0020±0.0008
	Pd_Pd1		2.9±0.6	3.02±0.03	0.007±0.001

	Pd_Pd2		4.6±0.9	3.4±0.0.1	0.007±0.001
Sample 1, 25°C fresh catalyst prior to NTP	Pd_O	5±1	3.5±0.3	2.01±0.01	0.001±0.001
	Pd_Pd1		2.6±1.7	3.03±0.02	0.003±0.005
	Pd_Pd2		4.1±2.5	3.43±0.01	0.003±0.004

Fitting parameters: S_0^2 = 0.85 as determined by the use of a Pd foil standard; Fit range 2.0 < k < 12, 1. < R < 3.6; number of independent points = 16

Table S3. σ^2 values calculated from EXAFS spectra measured for Pd/Al_2O_3 during cooling in air.

Temperature (°C)	σ^2 (Pd_O path)	σ^2 (Pd_Pd1 path)	σ^2 (Pd_Pd2 path)
37	0.0016±0.0006	0.007±0.001	0.007±0.001
51	0.0024±0.0007	0.007±0.001	0.007±0.001
101	0.0022±0.0006	0.009±0.002	0.008±0.001
308	0.0041±0.0007	0.013±0.003	0.010±0.002
405	0.0034±0.0007	0.015±0.004	0.013±0.003
500	0.0050±0.0009	0.017±0.005	0.013±0.003

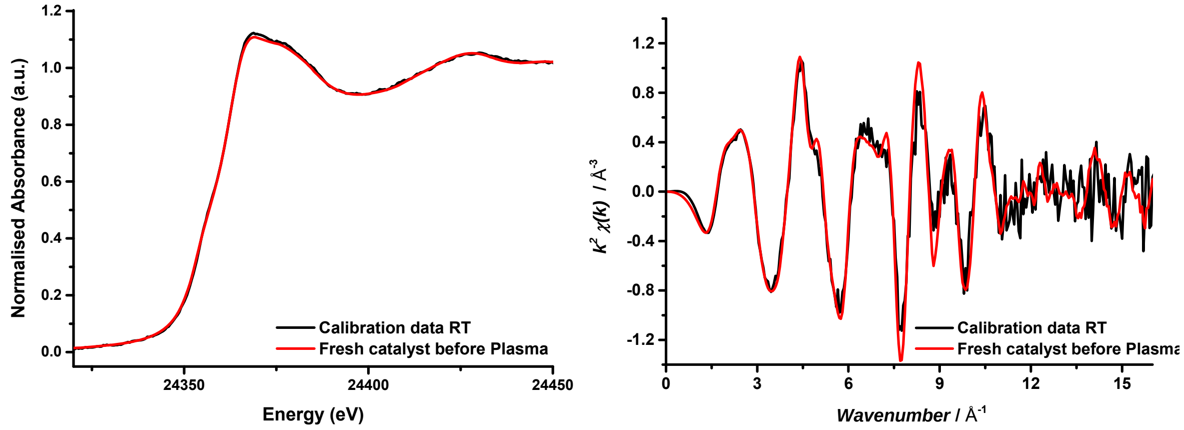

Figure S10 Comparison of sample 3 used for the calibration of σ^2 with sample 1 used for the NTP-CH_4 oxidation reaction (a) XANES spectra, (b) $k^2\chi$ data.

Effect of nanoparticle size and palladium loading on temperature induced by the NTP

A correlation between temperature effect of the plasma and nanoparticle size/palladium loading was investigated using a series of Pd/Al_2O_3 catalyst of different nanoparticle sizes, ranging from 1 nm – 4 nm and Pd loading (2 and 3 wt%). The σ^2 values obtained from the fit of the EXAFS data and the resulting estimates of the temperature are shown in Table S4. No obvious correlation between particle size or palladium loading and estimated temperature is observed, i.e. within error the temperatures measured for each sample are equal. This implies that the temperature is uniform throughout the whole nanoparticle and that the heating effect is not localised to the surface.

Table S4. EXAFS Fitting Parameters for Pd K-Edge spectra taken under CH_4 oxidation.

Conditions	Absorber-Scatterer	ΔE_0 (eV)	CN[c]	R_{eff} (Å)	σ^2	σ^2 (Plasma on data)	Estimated Temperature (°C)
[a]Sample 1 (2 nm)	Pd_O	8.0±0.8	4.2±0.1	2.020±0.006	0.0028(set)	0.0038±0.0004	179±49
	Pd_Pd1		4.9±0.3	3.058±0.007	0.0069(set)	0.010±0.001	
	Pd_Pd2		6.3±0.5	3.441±0.006	0.0072(set)	0.11±0.001	
[b]Sample 2 (4 nm)	Pd_O	7±1	4.0±0.2	2.02±0.01	0.0026(set)[e]	0.0036±0.0006	147±106
	Pd_Pd1		2.5±0.6	3.06±0.02	0.007(set)[e]	0.0097±0.002	
	Pd_Pd2		3.4±0.8	3.43±0.02	0.007(set)[e]	0.0095±0.002	
[c]Sample 3 (1.6 ± 0.3 nm)	Pd_O	8.0±0.8	4.2±0.2	2.023±0.005	0.0025±0.0006	0.0034±0.0003	138±18
	Pd_Pd1		5.7±0.8	3.059±0.006	0.0066±0.0009	0.0091± 0.0004	
	Pd_Pd2		6±1	3.446±0.001	0.005± 0.001	0.0083± 0.0005	
[d]Sample 4 (0.8±0.9 nm)	Pd_O	5±2	4.0±0.3	2.014±0.009	0.003±0.001	0.0035± 0.0004	150±46
	Pd_Pd1		3±1	3.04±0.01	0.006±0.003	0.009± 0.0001	
	Pd_Pd2		6±2	3.43±0.01	0.008± 0.003	0.011± 0.001	
Thermal CH_4 oxidation	Pd_O	-0.7±0.8	4.2±0.6	2.024±0.006	0.0025± 0.0006	0.0049±0.0008	507±152
	Pd_Pd1		4.2±0.6	3.058±0.006	0.0059±0.0008	0.017±0.003	
	Pd_Pd2		4.9±0.8	3.445±0.006	0.0054±0.0009	0.009±0.002	

[a]Fitting parameters: S_0^2 = 0.85 as determined by the use of a Pd foil standard; Fit range 3.3 < k < 10.4, 1. < R < 3.5; number of independent points = 11.3; [b]Fit range 3.3 < k < 10.4, 1. < R < 3.5; number of independent points = 11.5; [c]Fitting parameters: 1.0 < R <3.5, 3.3 < k < 14.1, number of independent points = 17.2; [d]Fit range 3.25 < k < 14.1, 1. < R < 3.5; number of independent points = 17.3; [e]the data quality of sample 2 was poor, the coordination numbers were refined whilst keeping the σ^2 values for the plasma off data set.

References

[1] D. E. Ashpis, M. C. Laun, E. L. Griebeler, ‘Progress Towards Accurate Measurements of Power Consumptions of DBD Plasma Actuators’ NASA/TM—2012-217449, AIAA–2012–0823;

[2] M. Newville, *J. Synchrotron Rad*. **2001**, *8*, 322-324;

[3] B. Ravel, M. Newville, *J. Synchrotron Rad.* **2005**, *12*, 537-541
